# Involvement of supralemniscal nucleus (B9) 5-HT neuronal system in nociceptive processing: a fiber photometry study

**DOI:** 10.1186/s13041-020-0553-1

**Published:** 2020-01-31

**Authors:** Shunpei Moriya, Akira Yamashita, Daiki Masukawa, Yuki Kambe, Junichi Sakaguchi, Honami Setoyama, Akihiro Yamanaka, Tomoyuki Kuwaki

**Affiliations:** 1 Department of Physiology, Kagoshima University Graduate School of Medical and Dental Science, Kagoshima, 890-8544 Japan; 2 Department of Molecular Pharmacology and Neurobiology, Yokohama City University Graduate School of Medicine, Yokohama, 236-0004 Japan; 3 Department of Pharmacology, Kagoshima University Graduate School of Medical and Dental Science, Kagoshima, 890-8544 Japan; 4 Research Institute of Environmental Medicine, Nagoya University, Nagoya, 464-8601 Japan

**Keywords:** Nociception, B9 5-HT cell group, Locus coeruleus, Ventral tegmental area, Fiber photometry, G-CaMP6, TPH2-tTA mice

## Abstract

Nociception is important perception that has harmful influence on daily life of humans. As to main pain management system, some descending pathways are called descending antinociceptive systems (DAS). As main pathways of DAS, it is well known that dorsal raphe (B6/B7) - rostral ventromedial medulla (B3) - spinal dorsal horn includes serotonergic system. However, possible role of supralemniscal (B9) serotonin (5-HT) cell group in pain management is still open question. In this study, we measured activities of B9 5-HT neuronal cell bodies and B9 5-HT neuron-derived axons located in the locus coeruleus (LC) and ventral tegmental area (VTA), which are also main players of pain management, using fiber photometry system. We introduced the G-CaMP6 in B9 5-HT neurons using transgenic mice carrying a tetracycline-controlled transactivator transgene (tTA) under the control of a tryptophan hydroxylase-2 (TPH2) promoter and site-specific injection of adeno associated virus (AAV-TetO(3G)-G-CaMP6). After confirmation of specific expression of G-CaMP6 in the target population, G-CaMP6 fluorescence intensity in B9 group and LC/VTA groups was measured in awake mice exposed to acute tail pinch and heat stimuli. G-CaMP6 fluorescence intensity rapidly increased by both stimuli in all groups, but not significantly reacted by nonnociceptive control stimuli. The present results clearly indicate that acute nociceptive stimuli cause a rapid increase in the activities of B9-LC/B9-VTA 5-HTergic pathways, suggesting that B9 5-HT neurons play important roles in nociceptive processing.

## Introduction

In clinical psychiatric medicine, clinicians take patients’ various perceptions into consideration. Nociception is important perception that has harmful influence on daily life of human [1]. Pain symptoms with some physical and mental disorders has increasingly become a major social problem. In the psychiatric field, pain symptom is frequently emerged in somatoform pain disorder [2], major depressive disorder [3], neuropathic pain [4], and sleep-related disorder [5]. As to drug therapy of these diseases, serotonin noradrenalin reuptake inhibitor (SNRI), selective serotonin reuptake inhibitor (SSRI), tricyclic antidepressant (TCA) and anticonvulsant are prescribed [6]. Monoaminergic system of central nervous system (CNS) is thought to be involved in the etiology of those diseases. As to main pain management system, there are some descending pathways [7, 8], which are called descending antinociceptive system (DAS). As main pathways of DAS, it is well known that the locus coeruleus (LC) – spinal dorsal horn circuit includes the noradrenergic system, periaqueductal gray - rostral ventromedial medulla (RVM) - spinal dorsal horn includes serotonergic (5-HTergic) system [9, 10]. Also, mesolimbic dopamine (DA) system affects regulating nociceptive activation [11, 12]. Studies have demonstrated that monoaminergic pathways play roles in processing nociceptive information by electrophysiological methods. Activation of LC NA neurons and RVM serotonin (5-hydroxytryptamine, 5-HT) neurons in response to nociceptive stimuli have been indicated by some electrophysiological studies [13, 14]. Also, ventral tegmental area (VTA) DA neurons play roles in regulating nociceptive information [12, 15, 16]. We recently certified that acute nociceptive stimuli rapidly increased the activities of LC noradrenalin (NA) neurons and RVM 5-HT neurons and VTA DA neurons in awake mice by using fiber photometry system [17, 18].

5-HT has extensive innervation in CNS [19] and subsets of the 5-HT cells are designed as B1-B9 groups in a caudal to rostral direction [20]. The dorsal raphe nucleus (DR: B7 and B6), median raphe nucleus (MR: B8 and B5), and RVM (B3) are well known as major parts. However, supralemniscal (B9) 5-HT cell group located just dorsal to the medial lemniscus is less known [21] and has hardly been studied. In this study, we focused on the possible contribution of B9 5-HT system in pain processing because B9 5-HT cells project to LC and VTA [22], which are important nuclei in pain processing (see above). We have demonstrated fiber photometry system [17, 18] for evaluating real time and cell type specific neuronal activity in awake mice with G-CaMP6 as a detector of Ca^2+^ concentration in the neuron of interest. This system has high temporal resolution (< sec) and unaffected by metabolism as in microdialysis, a different character from other electrophysiological and chemical methods. Our study reported that acute nociceptive stimuli increased the activity of LC NA neurons or the activity of VTA DA neurons by adopting fiber photometry system. Therefore, we thought that it is meaningful to appraise the activities of B9-LC 5-HTergic pathway and B9-VTA 5-HTergic pathway in response to acute nociceptive stimuli.

We first introduced the G-CaMP6 in B9 5-HT neurons using transgenic mice carrying a tetracycline-controlled transactivator (tTA) transgene under the control of a tryptophan hydroxylase-2 (TPH2) promoter and site-specific injection of adeno associated virus (AAV-TetO(3G)-G-CaMP6). We confirmed the specific expression of G-CaMP6 in B9 neuronal cell body and axon located at the targeted sites (LC and VTA) using an immunohistochemical method. We measured the Ca^2+^ signal of G-CaMP6 in these sites of awake mice while they were exposed to acute nociceptive stimuli.

## Material and methods

### Animals

**Fig. 1:**
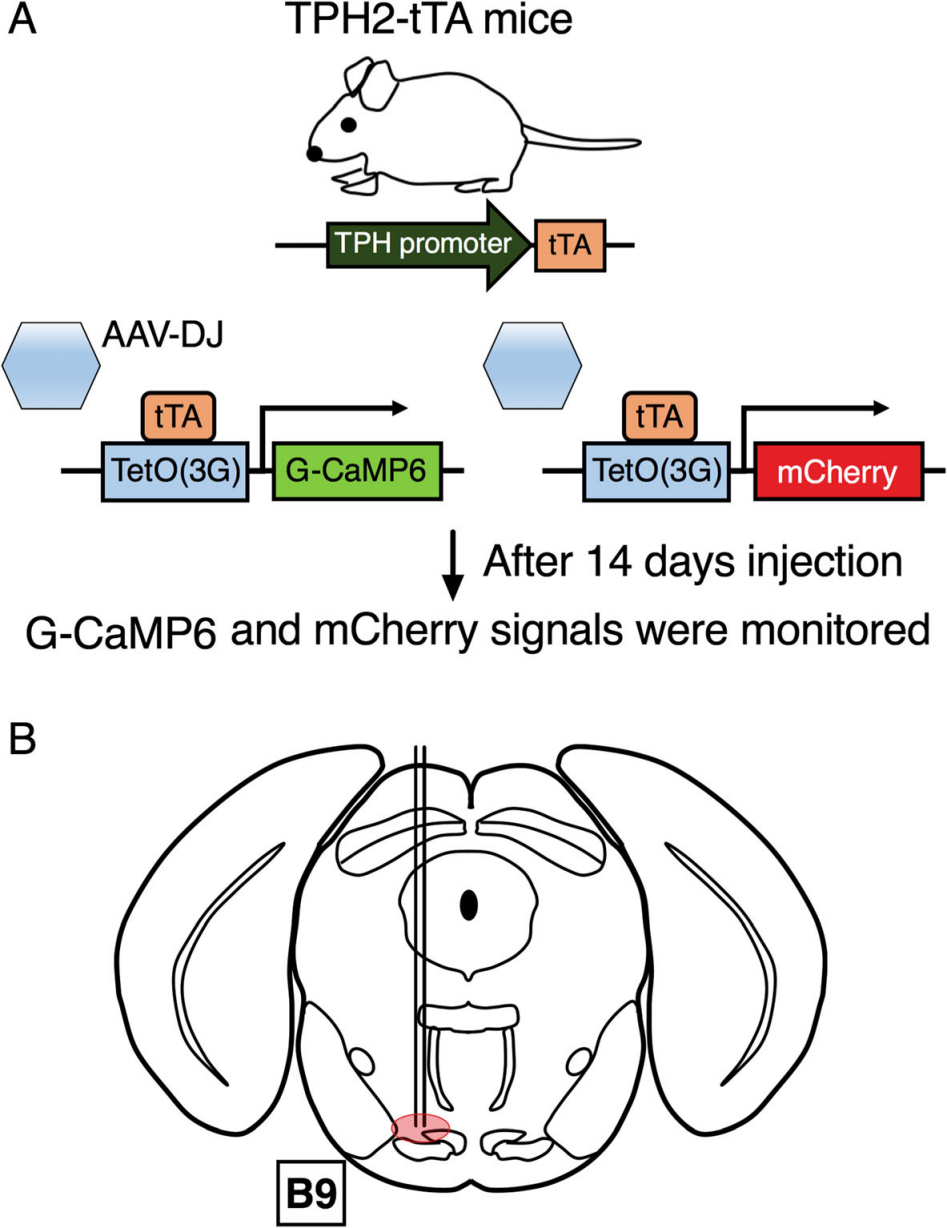
Development of serotonin neuron specific expression of G-CaMP6/mCherry using tet system. **a** TPH2-tTA mouse was injected with AAV-TetO- G-CaMP6/mCherry and was individually kept for 14 days before the experiment. **b** AAV was unilaterally injected into B9 site

The tryptophan hydroxylase-2 tetracycline-controlled transactivator (TPH2-tTA) transgenic mice were used [18, 23, 24] (Fig. 1a). We have already confirmed the specificity of TPH2-tTA expression in the previous report [24]. Ten to fourteen-week-old mice were used in this study. All mice were kept on a condition of 12 h light/dark cycle (7:00 AM to 7:00 PM), the temperature of 24 ± 1 °C, food and water were available ad libitum. All efforts were made to minimize animal suffering and discomfort; to reduce the number of animals used. All experimental procedures were performed in accordance with the National Institute of Health Guide for the Care and Use of Laboratory Animals and approved by the Institutional Animal Use Committee of Kagoshima University (MD17090).

### Stereotaxic AAV injection

AAV vector production was performed by AAV Helper-Free system (Agilent Technologies, Inc., Santa Clara, CA, USA); their purification was as previously described [17, 25, 26]. Mice were anesthetized with 2–3% isoflurane using a vaporizer for small animals and fixed with stereotaxic instrument (ST-7, Narishige, Tokyo, Japan) with an aid of supportive ear bar (EB-6, Narishige) of which touching surfaces to the animal were covered with local anesthetic jelly (lidocaine, 2% Xylocaine AstraZeneca). Both eyes were preserved with vaseline, head hair was shaved using an electric hair shaver, and cranial dura mater was cut open with small scissors. We slowly sucked up AAV into a glass micropipette (1B150F-3, World Precision Instrument, Inc., Sarasota, FL, USA), which was connected to a nitrogen pressure source through polyethylene tubing and to an injection manipulator (I-200 J, Narishige). In this study, AAV-TetO(3G)-G-CaMP6 (Serotype: DJ; 1 μl/injection, 4 × 10^13^ copies/ml) and AAV-TetO (3G)-mCherry (Serotype: DJ; 1 μl/injection, 6 × 10^12^ copies/ml) (Ohkura et al., 2012) (Fig. 1a) were unilaterally injected into B9 site (Injection site was from bregma − 4.36 mm, lateral + 0.38 mm left side, and ventral − 5.08 mm from the cranium) (Fig. 1b). After AAV injection, the micropipette was left in place for 10 min before being slowly withdrawn; mice were given an antibiotic, penicillin G (40,000 U kg-1) through subcutaneous injection. After operation, each mouse was individually kept for 14 days (2 weeks) in normal breeding conditions (as mentioned in Animals section) because it is needed for mice to recover and it takes about 2 weeks for G-CaMP6 or mCherry to fully express (Fig. 1a).

### In vivo fiber photometry system

**Fig. 2:**
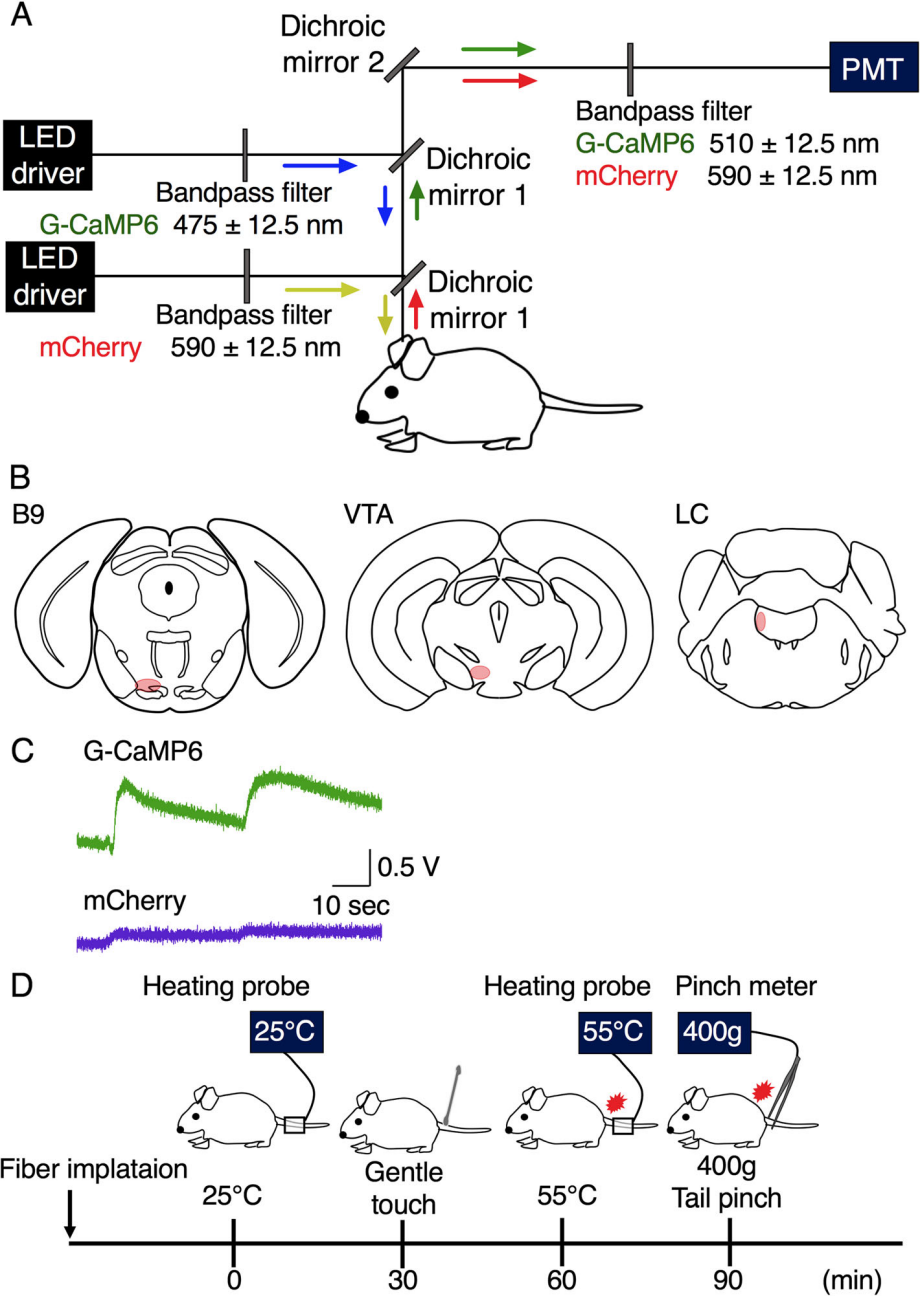
Experimental procedures. **a** Schematic representation of the fiber photometry system with two channels. **b** Target sites in this study: B9, LC and VTA. **c** The fluorescence signal intensity was abruptly increased when the position of the optical fiber tip was placed just above target site. **d** Schematic representation of the procedure of the recording. Fiber implantation was performed under isoflurane anesthesia. We set 3 h before the beginning of experimental sessions so that anesthesia would not affect experimental sessions. Total four stimuli (two kinds of acute nociceptive stimuli and two kinds of noninvasive control stimuli) were set in order from weaker stimulus to stronger stimulus as follows; the first is low temperature heat stimulus at 25 °C, the second is a gentle touch, the third is acute heat stimulus at 55 °C, and the last is acute mechanical tail pinch stimulus at the force of 400 g. Inter-stimulus interval was set as 30 min to reduce possible carryover effect from the previous stimulus

We showed fiber photometry system in previous reports [17, 18, 25, 26]. In this study, we adopted the fiber photometry system with two channels (Fig. 2a). In the first channel setting, the high-power LED driver (LEDD1B/M470F3, Thorlabs, Inc., Newton, NJ, USA) continuously produces blue excitation light (470 nm, 0.5 mW at the tip of the silica fiber) and the light passes through the excitation bandpass filter (475 ± 12.5 nm) and reflected by dichroic mirror-1; into a silica fiber (diameter: 400 μm, numerical aperture = 0.6). The same fiber detects and collects the green fluorescent signal of G-CaMP6. The signal passes through dichroic mirror-1 and reflected by dichroic mirror-2 and passes through the bandpass emission filter (510 ± 12.5 nm) and guided to a photomultiplier tube (PMTH-S1-1P28, Zolix Instrument, Beijing, China). At the second channel setting, the high-power LED driver continuously produces yellow excitation light (590 nm) and the light passes through the excitation bandpass filter (590 ± 12.5 nm) and goes forward as well. The same fiber detects and collects the red fluorescent signal of mCherry. The signal was forward and passed through the bandpass emission filter (607 ± 12.5 nm) and guided to another photomultiplier tube. First channel was adopted for detecting the neuronal activity and the second channel was used as an indicator of total stability of the fiber photometry system because mCherry fluorescence doesn’t reflect neuronal activity [26]. Both signals were digitized by an A/D converter (PowerLab8/35, ADInstruments Inc., Dunedin, New Zealand) and recorded by Labchart version-7 software (ADInstruments Inc.). Signals were collected at a sampling frequency of 100 Hz.

### Immunohistochemistry

To confirm AAV-induced expression of G-CaMP6 and mCherry in 5-HT neurons, after the experiments, mice were processed for immunostaining. Mice were deeply anesthetized with urethane (1.6 g/kg, i.p.) and transcardially perfused with 20 ml of phosphate buffered saline (PBS) and 20 ml of 4%-paraformaldehyde in PBS (Nacalai Tesque Inc., Kyoto, Japan). The brain was removed and post fixed in the same paraformaldehyde solution and soaked in 30% sucrose in PBS for 2 days. We formed serial 30 μm coronal sections including target sites (B9, LC, and VTA) with the cryostat (Cryotome FSE, Thermo Scientific, Yokohama, Japan). Every third section was adopted and floating immunohistochemical staining was performed. The sections were soaked in blocking solution (PBS containing 1% normal horse serum and 0.3% Triton-X) for 1 h at room temperature and incubated with anti-TPH antibody (AB1541, raised in sheep, EMD Millipore Corp., 1:1000) or anti-serotonin transporter (SERT) antibody (HTT-GP Af1400, raised in guinea pig, Frontier Institute, Hokkaido, Japan, 1:200) in blocking solution for overnight. The next day, the sections were washed three times with PBS and incubated with CF647 donkey anti-sheep IgG (20,284, Biotium, Inc., Fremont, CA, USA, 1:200) or CF647 donkey anti-guinea pig IgG (20,837, Biotium, 1:200) in PBS for 2 h in a dark box. In some sections in LC, VTA, B9 were treated with anti-tyrosine hydroxylase (TH) antibody (AB152, Millipore, raised in rabbit, 1:500) and visualized with CF647 donkey anti-rabbit IgG (20,047, Biotium).

After incubation, the sections were washed once with PBS and mounted on microscope slides (PRO-02, Matsunami, Osaka, Japan) and covered with microcover glasses (C024601, Matsunami). We observed and imaged the sections under the fluorescence microscope (BZ-X700, Keyence, Osaka, Japan), and analyzed the images with Adobe Photoshop CC (Adobe Systems, Inc., San Jose, CA, USA). G-CaMP6 and mCherry were visible without immunostaining.

### Acute nociceptive test

We applied two types of acute stress test showed in previous reports [17, 18]. We applied acute tail pinch stimulus using a pinch meter (PM-201, Soshin-Medic, Chiba, Japan) and acute heat stimulus using a heating probe (5R7–570, Oven Industries, Inc., Mechanicsburg, PA, USA). Pinch stimulus was attached to the root of the tail for three seconds with a force of 400 g and heat probe set at 55 °C was attached to the root of the tail for three seconds. We applied two noninvasive stimuli in the control group: gentle touch using a cotton stick and low temperature heat stimulus set at 25 °C using the same heating probe (5R7–570).

### Experimental protocol

In this study, we recorded G-CaMP6/mCherry green/red fluorescence intensity of B9 5-HT neuronal cell body and the axon located in LC/VTA (projecting sites of B9 5-HT neurons, Fig. 2b) to acute nociceptive stimuli. Each mouse was individually kept for at least 14 days after injection of AAV (Fig. 1a). The mouse was again anesthetized with 2–3% isoflurane using a vaporizer for small animals and fixed with stereotaxic instrument (ST-7) with an aid of supportive bar (EB-6) of which touching surfaces to the animal was covered with local anesthetic jelly (AstraZeneca). Following experiments were conducted in head-fixed condition. The head hair was shaved using electrical hair shaver; cranial dura mater was cut open with small scissors. A silica fiber was slowly implanted into the places just above B9 (bregma − 4.36 mm, lateral + 0.38 mm left, and ventral − 5.08 mm from the cranium), LC (bregma − 5.34 mm, lateral + 0.80 mm left, and ventral − 2.60 mm from the surface of the brain) and VTA (bregma − 3.15 mm, lateral + 0.50 mm left, and ventral − 4.15 mm from the surface of the brain) (Fig. 2b). We monitored the fluorescence signal intensity throughout fiber implantation and confirmed that the fluorescence signal intensity increased abruptly when the optimal position of the fiber tip was placed just above the target site (Fig. 2c). Open space around the optic fiber was covered with an ointment to avoid possible drying. After the fiber was fixed and positioned to the optimal position, anesthesia was turned off; each mouse recovered from anesthesia. We set 3 h before the beginning of experimental sessions so that anesthesia would not affect experimental sessions. The mice were divided into three groups; B9 group (n = 6), LC group (n = 6) and VTA group (n = 6). Experimental sessions of each mouse consist of two kinds of acute nociceptive stimuli and two kinds of noninvasive control stimuli (total four stimuli). To reduce the effect from the previous stimulus, we set inter-stimulus intervals at 30 min; their stimuli were set in order of weaker to stronger stimulus as follows; the first is low temperature heat stimulus at 25 °C, the second is a gentle touch, the third is acute heat stimulus at 55 °C; the last is acute mechanical tail pinch stimulus at the force of 400 g (Fig. 2d). After experiments, mice were euthanized and processed for immunostaining.

The definition of neuronal activity characteristic index was set as follows: F: averaged fluorescent signal intensity value for three seconds just before each stimulus and defined as 100%; ΔF: (maximum fluorescent signal intensity value during each stimulus) – F; onset latency: time from the start of stimulus to the time when the fluorescence signal intensity exceeded the maximum value during the baseline period; peak latency: time from the start of stimulus to the time when the fluorescence signal intensity reach the maximum value.

### Statistical analysis

Data analysis was conducted by two-way analysis of variance (ANOVA) with Sidak’s test for post hoc analysis. Two factors on ∆F/F were modality (mechanical vs. thermal) and intensity (nociceptive vs. gentle control). Factors on latency were modality (mechanical vs. thermal) and brain area (B9, LC, or VTA). Values are expressed as the mean ± standard error of the mean (S.E.M). Probability values less than (*p* < 0.05) were considered statistically significant. The analyses were performed using GraphPad Prism version 7 (GraphPad software, San Diego, CA, USA).

## Results

### Restricted expression of AAV-induced G-CaMP6/mCherry

**Fig. 3:**
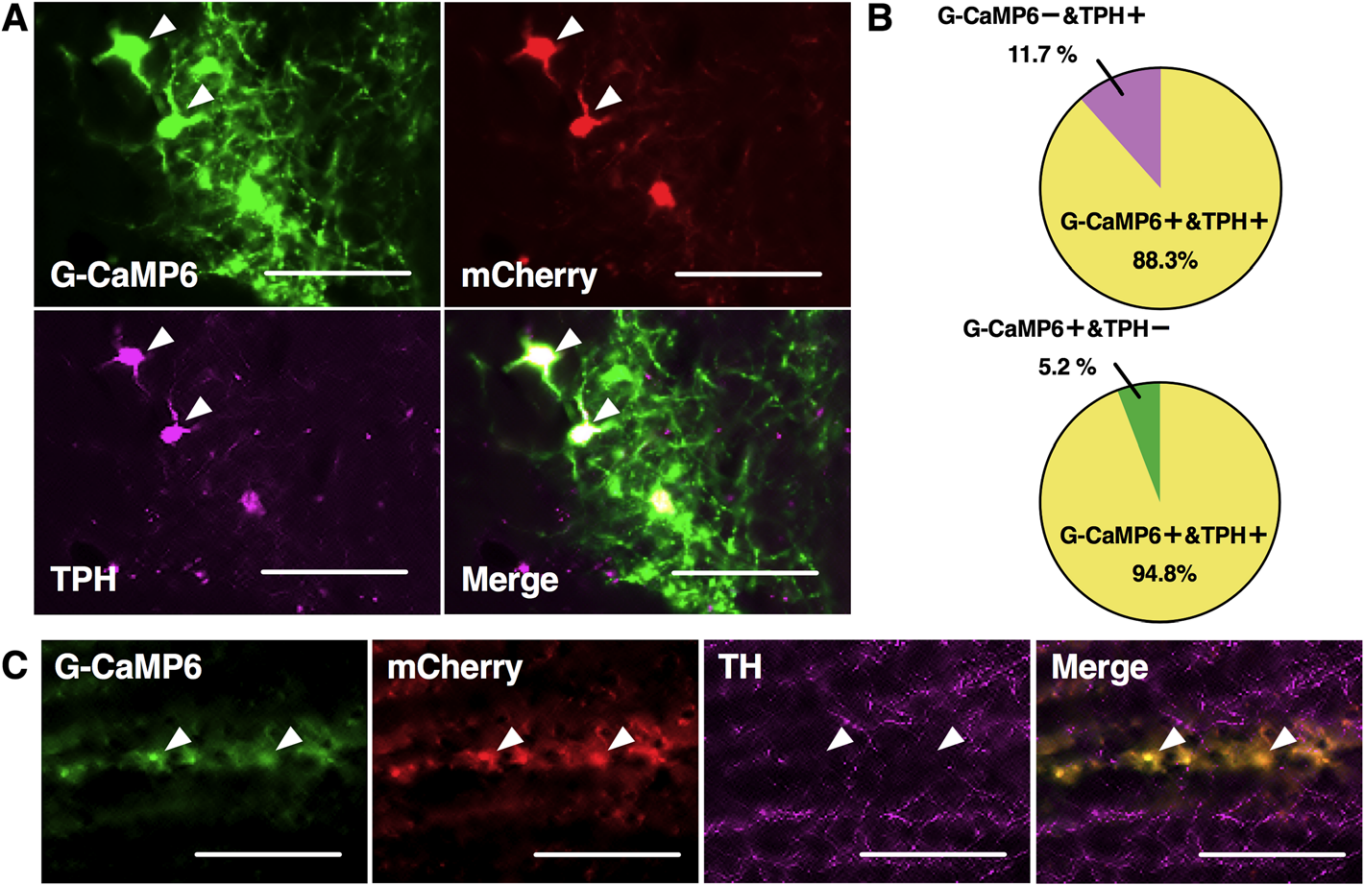
Specific expressions of G-CaMP6/mCherry in B9 area. **a** Fluorescence from G-CaMP6 (green) and mCherry (red) overlapped on TPH-positive cell soma (pink) in B9 area indicating specific expression of G-CaMP6/mCherry B9 5-HT neurons. Arrowheads show typical examples. **b** The percentage of G-CaMP6+ and TPH+ double positive cells in total TPH+ cells (upper) and the percentage of G-CaMP6+ and TPH+ double positive cells in total G-CaMP6+ cells (lower) (n = 6). **c** G-CaMP6/mCherry double positive cells did were not stained with anti-TH antibody immunostaining. Scale bar length:100 μm

**Fig. 4:**
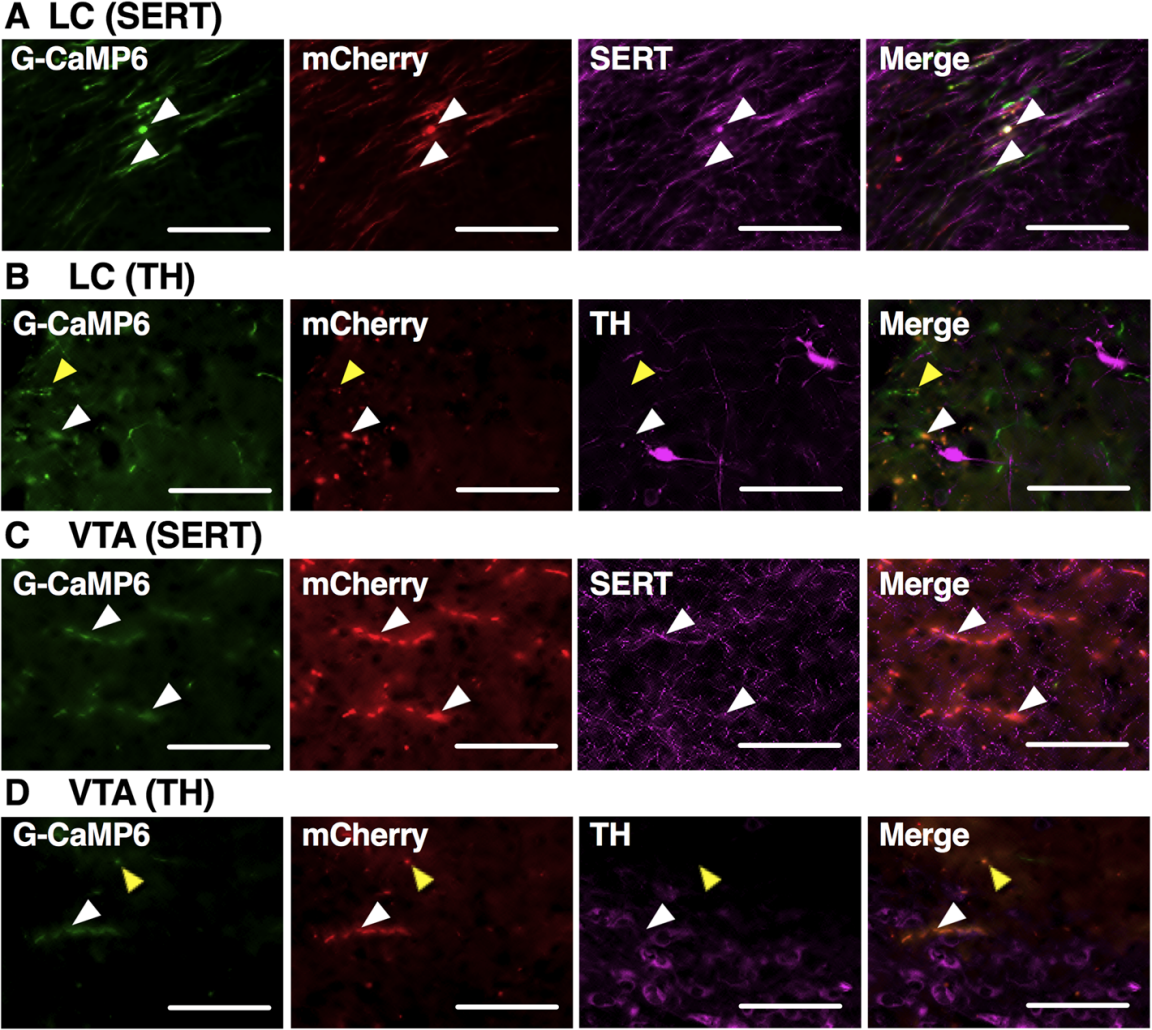
Expression of 5-HT fibers in LC and VTA. In LC (**a**, **b**) and VTA (**c**, **d**), triple positive axon fibers were confirmed (white rectangles). These fibers represent B9 5-HT-derived axons since AAV was locally injected into B9. G-CaMP6/mCherry positive soma and axons were hardly observed outside SERT positive structures. In LC (**b**) and VTA (**d**), G-CaMP6/mCherry double-positive fibers were found not only near TH positive cell body but also TH negative areas (yellow rectangles) within the nucleus, indicating B9 5-HT neurons project to not only catecholaminergic neurons (NA in LC and DA in VTA) but also other neurons in the target nuclei. Scale bar length:100 μm

Specific expressions of G-CaMP6/mCherry were confirmed in B9 5-HT neuronal soma (Fig. 3a, b). We found 23.3 ± 1.8 (n = 6, one representative slice per mouse) TPH-positive cells in the B9 and 88.3% of them also expressed G-CaMP6. All of the G-CaMP6 positive cells also expressed mCherry and 94.8% expressed TPH. Although anti-TPH antibody we used (AB1541) sometimes binds TH, distribution of anti-TH-positive structure in B9 did not overlap with that of G-CaMP6 (Fig. 3c). These expressions were also confirmed in B9 5-HT-derived axons located in LC (Fig. 4a and b) and VTA (Fig. 4c and d). G-CaMP6/mCherry positive soma and axons were hardly observed outside SERT positive structures (Fig. 4a and c). In LC and VTA, G-CaMP6/mCherry double positive fibers were found not only near TH positive cell body (white rectangles in Fig. 4b and d) but also TH negative areas (yellow rectangles) within the nucleus. Therefore, fluorescence was detected in specific manner in B9/LC/VTA sites.

### Effects of acute nociceptive stimulus on fluorescence intensity of G-CaMP6 and mCherry

We confirmed proper positioning of the silica fiber on B9, LC, and VTA by both physiological method (Fig. 2c) and histological method (Additional file 1: Figure S1).

**Fig. 5:**
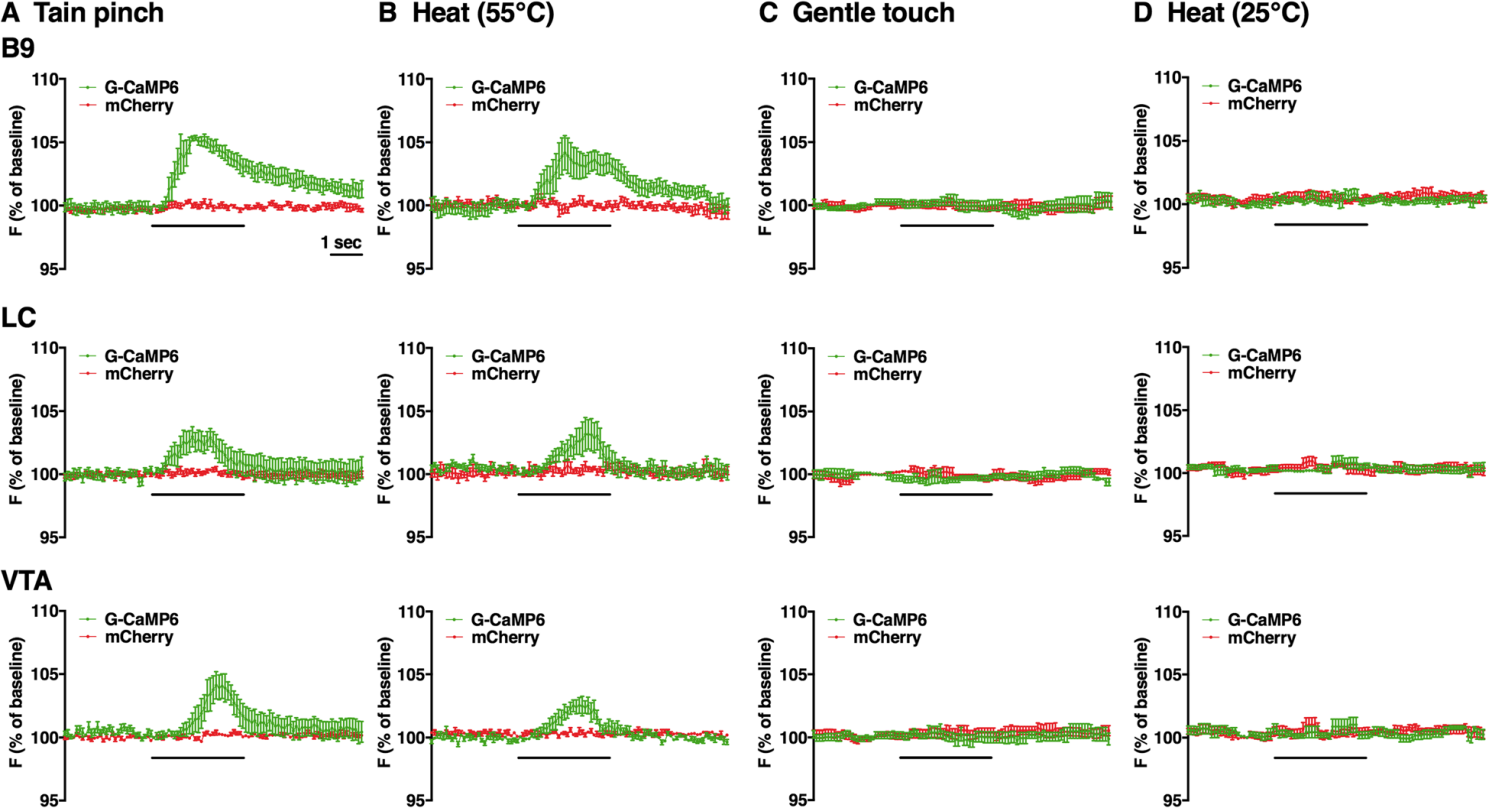
Averaged traces of the fluorescence intensity of G-CaMP6 and mCherry. **a** Tail pinch, **b** Heat, **c** Gentle touch, **d** Low heat. Recordings taken from B9, LC, and VTA (from top to bottom). Horizontal bar shows time of stimulation. Each trace shows an average in 6 animals. Vertical bars indicate S.E.M

**Fig. 6:**
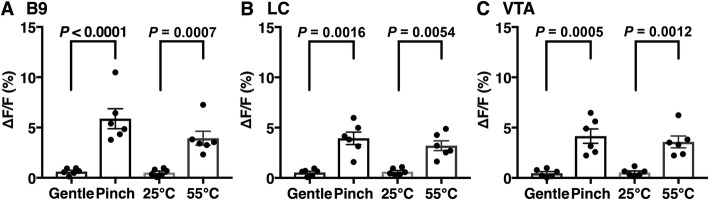
Effects of aversive and control stimuli on G-CaMP6 fluorescence intensity. (**a**) B9 5-HT neuronal soma, (**b**) B9 5-HT-derived axon in LC, and (**c**) B9 5-HT-derived axon in VTA. Values are expressed as the mean ± S.E.M (n = 6, each). Statistical analysis was conducted by two-way ANOVA with Sidak’s test for post hoc analysis. *P* values by the Sidak’s post hoc test are shown in the figure

Figure 5 shows the trace of G-CaMP6/mCherry fluorescence intensity associated with acute nociceptive stimulus (Fig. 5a-d). G-CaMP6 fluorescence intensity in B9 group and LC/VTA groups was rapidly increased by two acute nociceptive stimuli but not by non-nociceptive control stimuli. Two factor ANOVA revealed that the increase in G-CaMP6 fluorescence was significantly different between stimulus intensities (gentle vs. nociceptive) (B9 group: F(1, 5) = 31.1, *p* = 0.0026; LC group: F(1, 5) = 55.2, *p* = 0.0007; VTA group: F(1, 5) = 24.7, *p* = 0.0042). G-CaMP6 fluorescence in B9 also showed significant difference between mechanical and thermal stimuli (F(1, 5) = 21.9, *p* = 0.0054) whereas those in LC (F(1, 5) = 1.21, *p* = 0.3210) and VTA (F(1, 5) = 4.23, *p* = 0.0949) did not. Subsequent post hoc analysis revealed that there was significant difference between control vs. nociceptive stimulus in every combination of brain area and stimulus modality (Fig. 6). Note that ΔF/F values during non-nociceptive control stimuli did not exceed the fluctuation of fluorescence intensity during the baseline period; B9 group (1.12 ± 0.17%), LC group (1.47 ± 0.33%), VTA group (1.21 ± 0.23%).

On the other hands, mCherry fluorescent intensity in B9 group and LC/VTA groups was not significantly different between stimulation intensities (nociceptive vs. gentle) and between modalities (mechanical and thermal) (B9 group intensity: F(1, 5) = 0.3281, *p* = 0.5916; B9 group modality: F(1, 5) = 0.00104, *p* = 0.9755; LC group intensity: F(1, 5) = 0.1215, *p* = 0.7416; LC group modality: F(1, 5) = 0.5470, *p* = 0.4928; VTA group intensity: F(1, 5) = 0.0049, *p* = 0.9471, VTA group modality: F(1, 5) = 0.09759, *p* = 0.767). ΔF/F values during both non-nociceptive control stimuli and nociceptive stimuli did not exceed the fluctuation of fluorescence intensity during the baseline period; B9 group (1.27 ± 0.21%), LC group (113 ± 0.23%), VTA group (1.05 ± 0.18%).

**Fig. 7:**
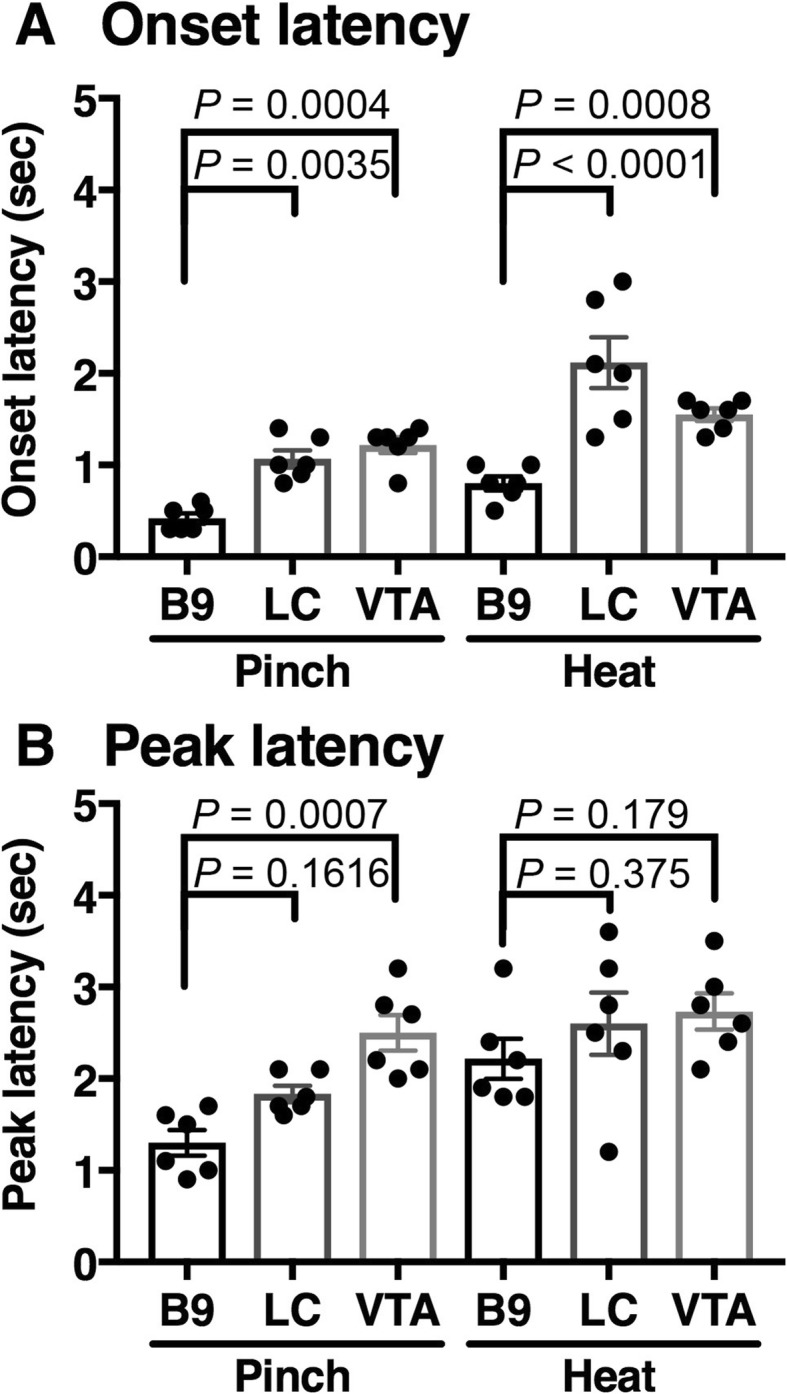
Characteristics of G-CaMP6 fluorescence response to aversive stimuli. **a** Onset latency; time from start of stimulus to the time when fluorescence signal intensity exceeded the maximum value during the baseline period. (**ab**) Peak latency; time from start of stimulus to the time when fluorescence signal intensity arrived at the maximum point. Values are expressed as mean ± S.E.M (n = 6, respectively). Statistical analysis was conducted by two-way ANOVA with Sidak’s test for post hoc analysis. *P* values by the Sidak’s post hoc test are shown in the figure

When comparing response characteristics, onset latency was significantly different among 3 brain areas (F(2, 15) = 57.19, *p* < 0.001) and also between modality (F(1, 15) = 19.77, *p* = 0.0005). Sidak’s multiple comparison revealed that onset latency in B9 was significantly shorter than that in LC and VTA in both pinch and heat stimuli (Fig. 7a).

Although 2-way ANOVA revealed significant difference in peak latency among 3 brain areas (F(2, 15) = 7.483, *p* = 0.0056) and between modality (F(1, 15) = 15.32, *p* = 0.0014), Sidak’s multiple comparison revealed that there was significant difference between B9 and VTA when pinch stimulus was applied and that there was no difference in other combinations (Fig. 7b).

## Discussion

The results of this study clearly demonstrated that acute nociceptive stimuli rapidly affected the activity of B9 5-HT neuronal cell bodies and B9 5-HT nerve axons located in LC and VTA in conscious mice adopting fiber photometry system. Recent tracer studies revealed B9-LC/B9-VTA 5-HT neuronal pathways [22]. B9 5-HT cell group comprises approximately 20% of the total mesopontine 5-HT neurons [21, 27], nevertheless has been much less studied compared to the wealth of studies on the DR, MR, and RVM groups. To our knowledge, our data using the fiber photometry system are the first report that measured the activities of B9 5-HT neurons during aversive stimuli and that showed possible role of B9 5-HT neurons in pain processing.

**Fig. 8:**
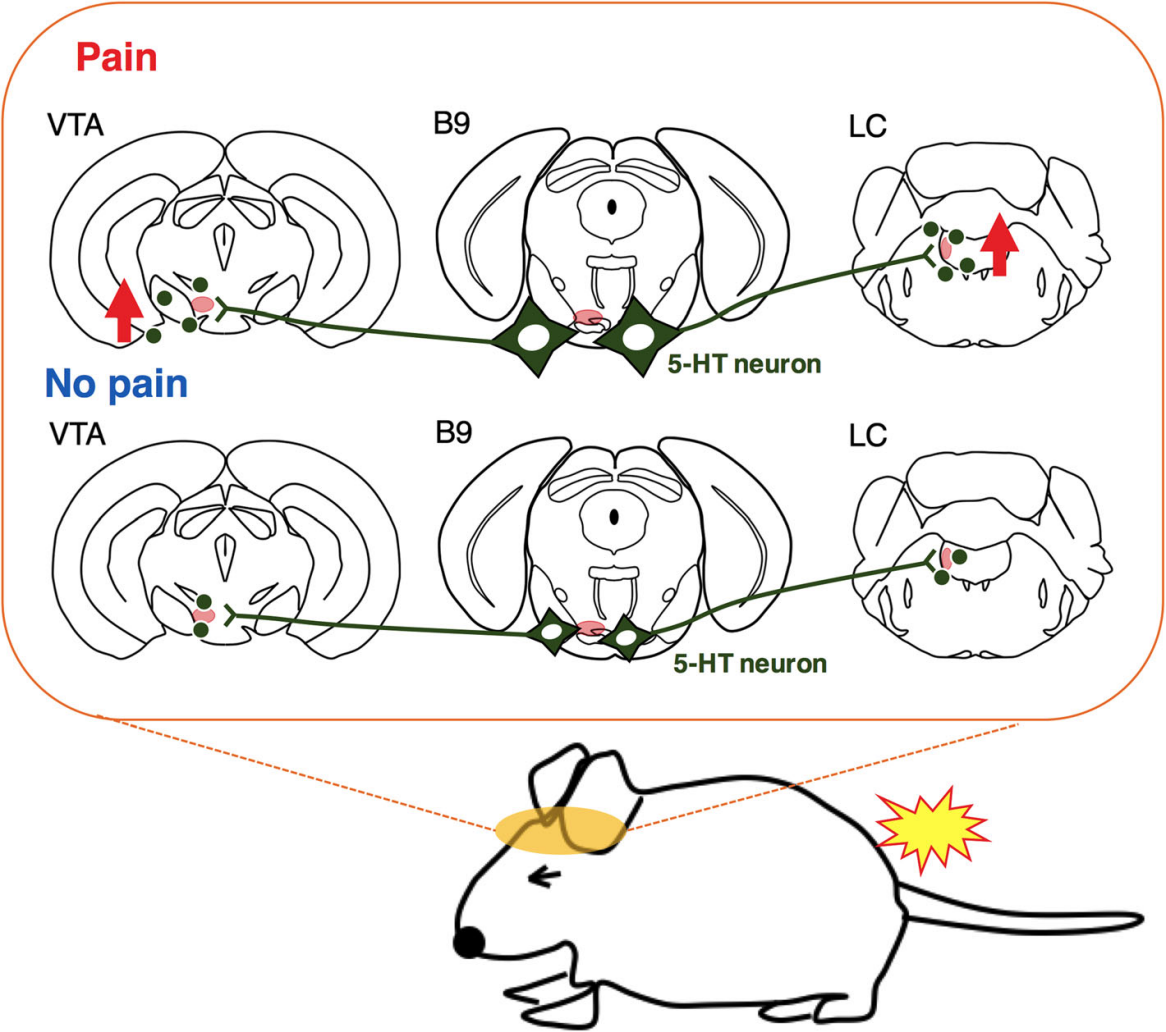
Schematic explanation of possible contribution of B9 5-HT neurons in pain processing

In addition, this is the first report that measured the activities of B9 5-HT nerve axons located in LC and VTA. The present results showed that the activity of B9-LC 5-HT pathway and B9-VTA 5-HT pathway were rapidly increased by acute nociceptive stimuli. The results of onset latency showed that in B9 was significantly shorter than those in LC or VTA in both pinch and heat stimuli (Fig. 7a). This result was in line with our hypothesis that the activities of B9 5-HT neuronal soma propagate to LC and VTA through B9 5-HT-derived axons (Fig. 8). Our previous studies using fiber photometry system showed that acute nociceptive stimuli rapidly increased the activities of LC NA neurons and VTA DA neurons [17, 18]. Some studies have reported the activation of LC NA neurons using microdialysis [14] or electrophysiological recording [28, 29]. Other studies have reported that nociceptive stimuli affected mesolimbic DA system [30, 31] and mesocortical DA system [32, 33]. In this regard, it is considered that B9 5-HT neuronal projection to LC affected the activity of LC NA neurons in pain processing system of DAS; in a similar manner, B9 5-HT neuronal projection to VTA affected the VTA DA neurons. This notion is supported by our histological examination showing the close location of B9 5-HT axon near the NA neurons in LC (Fig. 4b) and the DA neurons in VTA (Fig. 4d). Although we have revealed possible flow of pain information from B9 to LC and VTA, we need more studies to reveal physiological importance and impact of this pathway in pain regulation.

We confirmed the expression of G-CaMP6/mCherry in B9 5-HT neurons in TPH2-tTA mice injected with AAV-tetO-GCaMP6/mCherry by immunohistochemical method. In a previous study, we showed the expression of G-CaMP6/mCherry in RVM/DR 5-HT neurons in TPH2-tTA mice using the same AAV [18]. Our present results coincide with the previous studies showing dense collections of 5-HT cells in B9 [21, 34]. Taken together, our method using AAV seemed applicable to study activity of any 5-HT neurons in the CNS.

Emerging evidence has pointed to anatomical and functional heterogeneity within the brainstem 5-HTergic cell groups [35, 36]. Nevertheless, the function of B9 5-HT neurons has been largely unknown, except for studies reporting that B9 5-HT neurons played prominent roles in shaping aggression [37] and regulating affect and stress responsivity [36, 38]. DAS, including LC, is considered to be involved in pain-emotion symptoms [28, 39] and VTA involved in mood symptom [40, 41] and fear [42]. An anatomical study demonstrated that B9 5-HT neurons project to the hypothalamus, cortex, and hippocampus that are related to some psychiatric symptoms [43]. Taking these observations together, our results suggest that B9-LC/B9-VTA 5-HT neuronal pathways may be related to pain-emotion symptoms.

In clinical psychiatric medicine, the center of therapeutic strategy is drug therapy and many psychiatric-related drugs affect the state of monoamines in CNS. SNRI, SSRI and TCA for pain treatment act on synapses of 5-HT neurons in CNS and it takes at least a few weeks to relieve pain symptoms [44]. In case of patients in the psychiatric field with pain symptoms receiving these drug therapy, the state of neuronal activity in a subset of 5-HTergic cell groups including B9 can be affected. This possibility seems worth testing in the future study.

Limitations in this study are as follows. We did not adopt psychiatric-related drugs, therefore did not appraise how these drugs affect the activities of B9 5-HT neurons. Also, our protocol is only acute nociceptive system. Therefore, in the future, the protocol including psychiatric-related drugs and chronic nociceptive system will be adopted. Fiber photometry system measures activities of ensemble average of the labeled neurons but not single unit activity. Combination of advantages of multiple methodologies are needed.

In conclusion, the results of this study suggest that acute nociceptive stimuli cause a rapid increase in the activities of B9-LC/B9-VTA 5-HTergic pathways and suggest that B9 5-HT neurons play important roles in nociceptive processing in the CNS.

## Supplementary information

**Additional file 1: Figure S1.** Confirmation of fiber implantation tracking. Fiber track was located just above B9, LC and VTA.

## Data Availability

All data in this study are available upon requests.

## Abbreviations

5-HTergic: Serotonergic; 5-hydroxytryptamine, 5-HTserotonin
AAV: Adeno associated virus
ANOVA: Analysis of variance
CNS: Central nervous system
DA: Dopamine
DAS: Descending antinociceptive system
DR: Dorsal raphe nucleus
LC: Locus coeruleus
MR: Median raphe nucleus
NA: Noradrenalin
PBS: Phosphate-buffered saline
RVM: Rostral ventromedial medulla
S.E.M: Standard error of the mean
SERT: Serotonin transporter
SNRI: Serotonin noradrenalin reuptake inhibitor
SSRI: Selective serotonin reuptake inhibitor
TCA: Tricyclic antidepressant
TH: Tyrosine hydroxylase
TPH2: Tryptophan hydroxylase-2
tTA: Tetracycline-controlled transactivator transgene
VTA: Ventral tegmental area
