# Supplementary figures and images for: Involvement of supralemniscal nucleus (B9) 5-HT neuronal system in nociceptive processing: a fiber photometry study
Source: Mol Brain. 2020 Jan 31;13:14. doi: 10.1186/s13041-020-0553-1 (PMC6993514; doi:10.1186/s13041-020-0553-1)

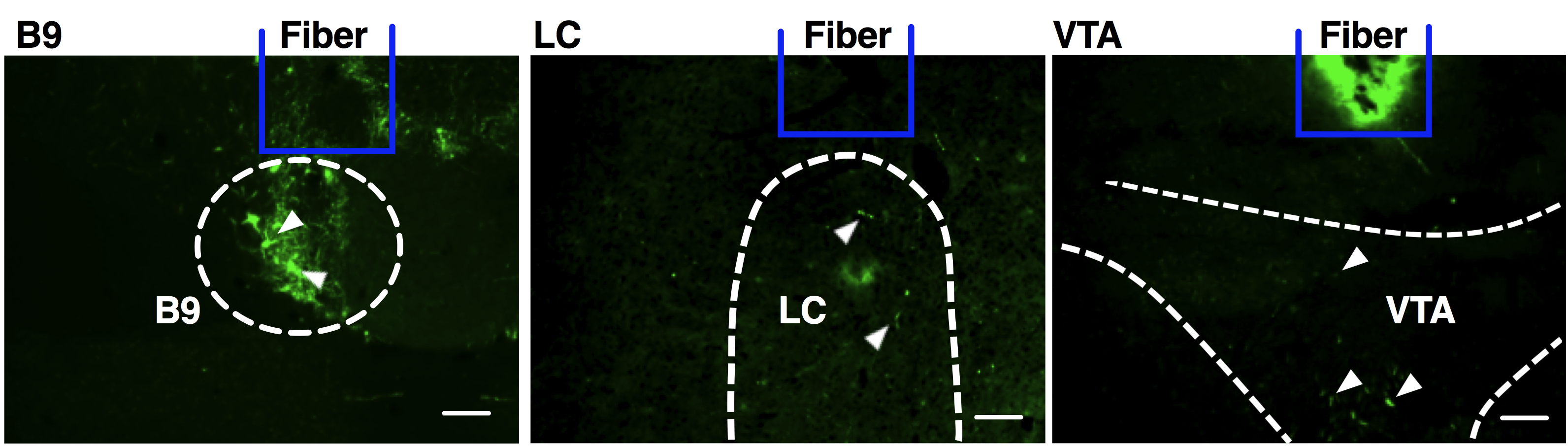

Supplement: Supplementary file 1 — Additional file 1: Figure S1. Confirmation of fiber implantation tracking. Fiber track was located just above B9, LC and VTA. [file 13041_2020_553_MOESM1_ESM.tiff]
